# Bacterial community structure of electrogenic biofilm developed on modified graphite anode in microbial fuel cell

**DOI:** 10.1038/s41598-023-27795-x

**Published:** 2023-01-23

**Authors:** Bahaa A. Hemdan, Gamila E. El-Taweel, Sunandan Naha, Pranab Goswami

**Affiliations:** 1 Water Pollution Research Department, Environmental Research and Climate Change Institute, National Research Centre, 33 El-Bohouth St., Dokki, 12622 Giza Egypt; 2 Department of Biosciences and Bioengineering, Indian Institute of Technology Guwahati, Guwahati, 781039 India

**Keywords:** Bioinformatics, Biological models, Electrophysiology, Biocatalysis, Environmental biotechnology

## Abstract

Formation of electrogenic microbial biofilm on the electrode is critical for harvesting electrical power from wastewater in microbial biofuel cells (MFCs). Although the knowledge of bacterial community structures in the biofilm is vital for the rational design of MFC electrodes, an in-depth study on the subject is still awaiting. Herein, we attempt to address this issue by creating electrogenic biofilm on modified graphite anodes assembled in an air–cathode MFC. The modification was performed with reduced graphene oxide (rGO), polyaniline (PANI), and carbon nanotube (CNTs) separately. To accelerate the growth of the biofilm, soybean-potato composite (plant) powder was blended with these conductive materials during the fabrication of the anodes. The MFC fabricated with PANI-based anode delivered the current density of 324.2 mA cm^−2^, followed by CNTs (248.75 mA cm^−2^), rGO (193 mA cm^−2^), and blank (without coating) (151 mA cm^−2^) graphite electrodes. Likewise, the PANI-based anode supported a robust biofilm growth containing maximum bacterial cell densities with diverse shapes and sizes of the cells and broad metabolic functionality. The alpha diversity of the biofilm developed over the anode coated with PANI was the loftiest operational taxonomic unit (2058 OUT) and Shannon index (7.56), as disclosed from the high-throughput 16S rRNA sequence analysis. Further, within these taxonomic units, exoelectrogenic phyla comprising *Proteobacteria*, *Firmicutes*, and *Bacteroidetes* were maximum with their corresponding level (%) 45.5, 36.2, and 9.8. The relative abundance of *Gammaproteobacteria*, *Clostridia*, and *Bacilli* at the class level, while *Pseudomonas*, *Clostridium*, *Enterococcus*, and *Bifidobacterium* at the genus level were comparatively higher in the PANI-based anode.

## Introduction

The bio-based processes used for wastewater management involve lesser operating costs and simpler operations than their chemical and physical counterparts^1^. Efforts are on to improve efficiency and couple value-addition benefits to the bio-based treatment processes to realize their full potential for real-world applications^2^. Transforming organic compounds in wastewater into valuable derivatives using bioelectrochemical systems (BESs) has drawn advancing attraction due to its prospect in miscellaneous implementations^3^. Microbial fuel cell (MFC) is an attractive addendum to this biotechnological venture for their potential to break down the biodegradable organic compounds that exist in wastewater bodies using electroactive microbes and simultaneously generate bioelectrical power through bioelectrochemical transformation strategies^4^. The kingpin of this conversion process is the natural microbial populations in the wastewater environment that colonize the MFC electrodes as biofilm and initiate the conversion process through their biocatalytic activities^5,6^. However, this bioelectrocatalytic conversion process of the complex organic substances present in the wastewater through the naturally evolved bacterial biofilm is prolonged and not competent enough to cope with the waste accumulation dynamics in open environment conditions. One critical issue that evokes this challenge is the sluggish formation of the electrogenic biofilm over the anodic surface. Therefore, the induction of microbial biofilm electrophoresis on the electrode surface is an important research area in MFC-based bioprocess technology^7^.

A plethora of scientific reports are available on developing electrogenic microbial biofilm on electrode surfaces for harvesting power in MFC^8,9^. Electrogens are electrochemically active microbes, most commonly bacteria, which produce electrical energy in an MFC setup by degrading organic compounds and transferring the generated electrons to an electrode^10,11^. The biofilm formation of these electrogens on the electrode (mostly anode) surface is a prerequisite for harvesting sufficient metabolic electrons from the oxidation of organic compounds in the wastewater for generating desired power in MFCs^12,13^. Different strategies have been investigated to create bacterial biofilm and improve electrical power in MFC, such as screening electrodes and coating materials over electrodes^14,15^, chemical coupling of biofilm with the base electrode^16^, nanofabrications^17^, and screening environmental waste to fabricate electrodes^18^. Among electrode materials, carbon-based materials are emerging as promising electrodes to enhance the electrochemical performance of the biofilm^19,20^.

Furthermore, some investigations on composite-based electrode materials were documented, including graphite/metal, carbon cloth/metal, carbon nanotubes/metal, and many other polymeric composites^21,22^. Most of these studies explored the impact of anode materials on the electrical performance of the MFC. Generally, these efforts have contributed to incremental progress on the subject. However, for the rational design of BFCs for practical applications, a comprehensive understanding of the bacterial community of the biofilm is essential^23^.

The main objective of this investigation is to investigate the bacterial community structure of the electrogenic biofilm developed on the anodic surface of a locally designed MFC system fed with activated sludge as a fuel source and bacteria. Using the graphite material generally employed as the primary electrode, the formation of electrogenic biofilm over the anode surfaces after electrode modification was scrutinized with various utilized conductive composites to screen the best support materials for biofilm development. We devoted the plant mixture with high carbohydrate and protein content as a coating composite was operated for the immediate induction of bacterial biofilm over the modified graphite anode. Microbial community structures at phylum, class, and species levels in the biofilm were analyzed using high-throughput 16S rRNA gene sequencing analysis. A detailed description of the results on the bacterial community profiles of surface-modified graphite anodes and the associated electrical performance of MFCs are described in this paper.

## Materials and methods

### Chemicals and reagents

Polyaniline (PANI), carbon nanotubes (CNTs), reduced graphene oxide powder (rGO), and proton exchange membrane (PEM: Nafion 117) were purchased from Sigma–Aldrich. Luria Bertani (LB) agar medium was purchased from Himedia Labs (India). Potato powder containing 15–20% carbohydrate was prepared by crushing the cleaned potato in a mixer grinder, then extracting the fine paste into a glass Petri dish. The paste was then calcined in a hot air oven overnight at 60 ± 5 °C, and the resulting fine powder was cooled to room temperature. Soybean powder containing 36–58% protein was prepared from raw dried soybeans purchased from the local market. Dried soybeans were properly crushed using a mixer grinder to the finest powder form. Finally, the Potato and Soybean powders were mixed at 1:1 to produce a homogeneous Potato-Soybean (plant) powder and then stored at 4 °C for further use. Deionized water (18.2 MΩ cm) from Millipore Co. was used throughout the experiment. The study of collection of plants/plant material, are in compliance with relevant institutional, national, and international guidelines and legislation. All reagents used were of analytical grade and used as received without further purification.

### Preparation of electrodes

Graphite plates with dimensions (2.0 cm × 2.0 cm × 0.3 cm) were employed as the conductive anodic electrodes. Three different anodes were fabricated by coating with the composite materials on the graphite electrodes as follows: S1: control (no coating); S2: composite suspension of plant powder and rGO (soybean: potato: rGO at a ratio of 1:1:0.5 w/w); S3: composite suspension of plant powder and PANI (soybean: potato: PANI at a ratio of 1:1:0.5 w/w); S4: composite suspension of plant powder and CNTs (soybean: potato: CNTs at a ratio of 1:1:0.5 w/w). Non-platinized gas diffusion electrodes were applied as air cathodes and were constructed at VITO (Belgium), as explained previously^24^. The electrodes were assembled in an MFC setup in a configuration described in the next section. Before MFC operation, the prepared anode electrodes were placed in a falcon tube containing 50 mL of domestic sludge collected from the sewage treatment plant, IIT Guwahati, Assam, India. The tubes were kept in an incubator at 35 °C ± 2 °C for 30 days to induce biofilm growth on the anode.

### MFC configuration

**Figure 1:**
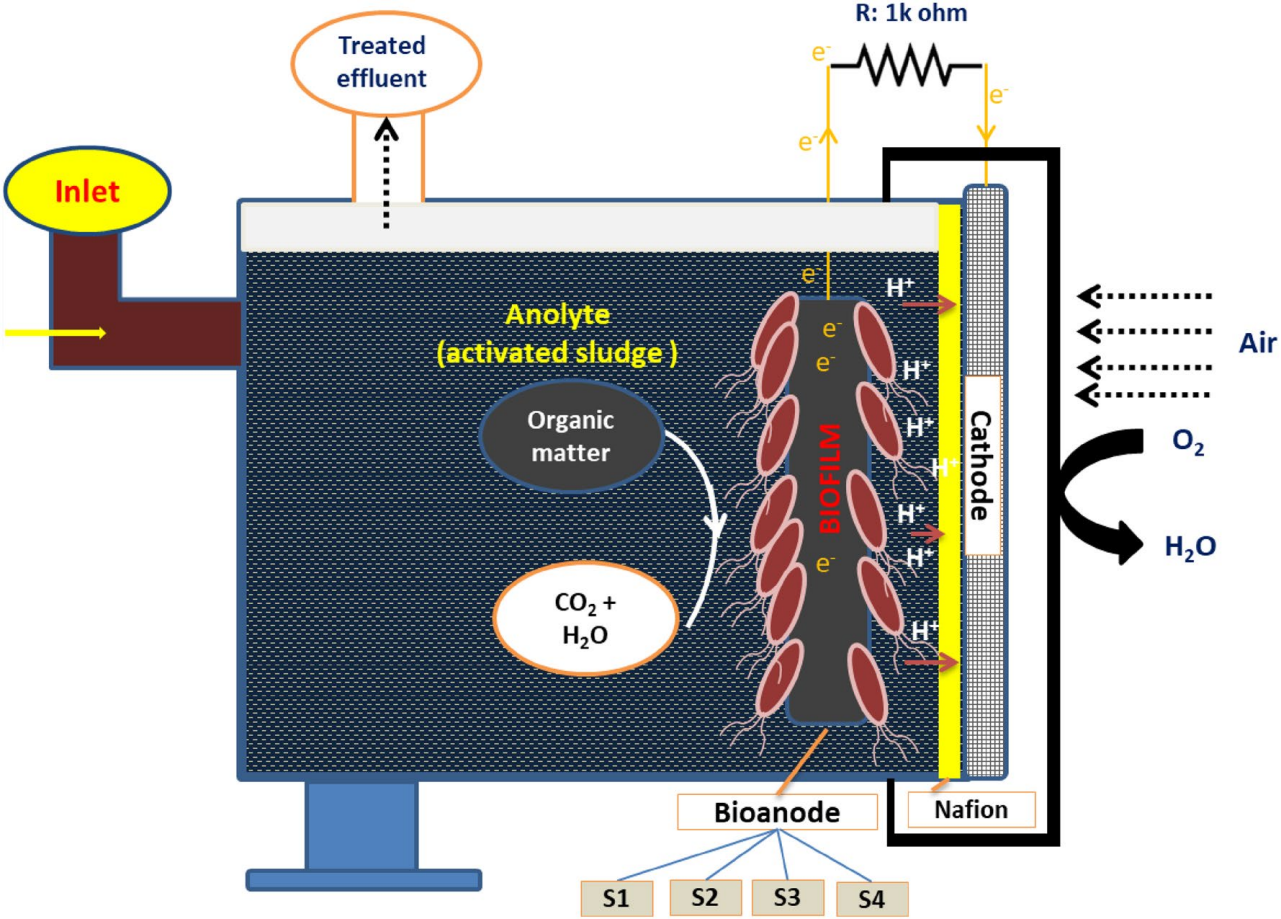
Configuration of the fabricated MFC system with a chamber. The rectangular-shaped chamber was made of polyacrylic plastic with a total liquid volume of 50 mL. The dimension (length × width × height) of the anodic chamber was 80 × 80 × 30 mm.

A single-chambered air–cathode MFC with a total working volume of 50 mL was constructed (Fig. 1). The anolyte in the anodic chamber was prepared by diluting the activated sludge with a phosphate buffer solution of pH 7.61 at a ratio of 4:1.

### Operation of MFC bioreactors

The MFC was operated using 50 mL activated sludge diluted with PBS at a specific ratio of sludge: PBS (4:1) in the anodic chamber without using any additional carbon and fuel source. The initial log count of bacterial cell densities was 10^7^ CFU/mL. The fabricated anodes, as mentioned above, were immersed in the anodic chamber. The anoxic condition in the anodic chamber was initiated by purging argon gas for 10 min following inoculation of anolyte before operating the MFC. Upon stabilization of the open-circuit potential (OCP), an external load (1 kΩ) was connected through a copper wire to draw the current from the MFC. The MFCs were operated for 45 days in a batch mode at 25 °C. The current produced by MFCs was recorded every 10 min by a data acquisition system (Agilent 34972 A LXI, USA)^25^.

### Investigation of anodic biofilm

#### Estimation of cell viability

The biofilm cell populations developed over the anode surfaces were monitored at regular two days intervals by counting the viable biofilm cells using the agar plating technique. The biofilm formed on the anodic electrode surface was washed three times with saline water, and then detached from the cells from the surface area using a sterile swab^26^. The collected swaps were placed in a 10 ml saline water glass test tube. The tubes were then agitated using vortex for 10 min to detach the swaps' biofilm cells. The cell viability assay was performed by transferring one ml of the detached biofilm cell suspension into sterile agar dishes containing 10–15 ml of LB agar at 35 °C. The plates were then placed in an incubator at 35 °C overnight to grow the bacterial colonies on the agar surface^27^.

#### Field emission scanning electron microscopy (FESEM) studies

Morphological aspects of the biofilm developed over anodes were examined using FESEM (Make: Zeiss, Model: Sigma) with 3 keV EHT, 50 mm aperture. Before imaging, the biofilm-coated electrodes were fixed with 2.5% glutaraldehyde solution for 4 h of retention time and washed using low ionic 20 mM potassium phosphate buffer, pH: 7.3 (PBS). Afterward, the electrodes were dehydrated using a 10–100% gradient alcohol series for 30 min for each stage with very gentle periodic agitation and then dried thoroughly^28^. The desiccated samples were vacuum dried, mounted onto stubs, sputtered with gold, and then captured images^29^.

### Microbial community analysis

#### Biofilm sample collection and preparation

At the end of the experimental process (45 days), the anode electrodes were withdrawn from the MFC reactors and then carefully washed under running water to dismiss any adhered debris. A sterile scalpel was employed to scrape the microbial biofilm that was developed over the anode electrode. The scraped anodic biofilm was deposited in a sterile 50 ml tube containing 10 ml of 50 mM PBS. Final biofilm samples were assigned for DNA extraction.

#### DNA isolation

The total genomic DNA of the collected biofilm samples was extracted using a PowerSoil DNA isolation kit (MoBio Laboratories Inc., USA) following the manufacturer's protocol. The intensity and purity of the isolated DNA extracts were examined by measuring absorbance at λ_260nm_ and λ_280nm_ using NanoDrop 8000 spectrophotometer. The isolated DNA of all biofilm samples was maintained at − 80 °C prior to downstream analyses^30^.

#### PCR reaction

The V3–V4 hyper-variable regions of the bacterial 16S rRNA gene were amplified using a thermocycler PCR system with a pair of universal bacterial primers, which were as follows: 16SrRNAF: (5′-GCCTACGGGNGGCWGCAG-3′) and 16SrRNAR: (5′-ACTACHVGGGTATCTAATCC-3′). PCR amplification was conducted using the following thermal-cycling program: 3 min of initial denaturation at 95 °C, 30 s for annealing at 55 °C, and 45 s for elongation at 72 °C with a final extension of 10 min. The resulting PCR product was resolved by adding 3 µl of PCR product into 1.2% agarose gel electrophoresis for ~ 60 min or till the samples reached 3/4th of the gel^31^.

#### Next-generation sequencing (NGS)

NGS was performed using MiSeq Illumina sequencing technology, focusing on the V3–V4 hyper-variable regions of the 16S rRNA gene to explore the microbial population dynamics in the anodic biofilm. The amplicon library was prepared using Nextera XT Index Kit (Illumina inc.) according to the 16S Metagenomic Sequencing Library Preparation protocol (Part # 15044223 Rev. B)^8^. Primers for amplifying the specific region were designed and synthesized at Eurofins Genomics Lab, India. The QC passed amplicons with the Illumina adaptor were amplified using i5 and i7 primers that add multiplexing index sequences and standard adapters required for cluster generation (P5 and P7) as per the standard Illumina protocol. The Illumina overhang adapter sequences were as follows: Forward overhang: 5′ (CGTCGGCAGCGTCAGATGTGTATAAGAGACAG) and Reverse overhang: 5′ (GTCTCGTGGGCTCGGAGATGTGTATAAGAGACAG). The amplicon libraries were purified by AMPure XP beads and quantified using Qubit Fluorometer. The amplified libraries were analyzed on the 4200 Tape Station system (Agilent Technologies) using D1000 Screen tape per manufacturer instructions. Equimolar quantities of all samples were pooled into one tube^32^. The amplicon library pool sequencing was performed on the Illumina MiSeq platform (Eurofins Genomics India Pvt. Ltd.).

### Bioinformatics analysis

FASTQC purified the Paired-end read data. High-quality clean reads were obtained using Trimmomatic v0.38 after removing adapter sequences, ambiguous reads (reads with unknown nucleotides “N” larger than 5%), and low-quality sequences (reads with more than 10% quality threshold (QV) < 20 Phred score) along with a sliding window of 10 bp and a minimum length of 100 bp. The single-end reads were merged using the FLASH (v1.2.11) software tool. The high-quality paired-end reads were analyzed using bioinformatics workflow Quantitative Insights Into Microbial Ecology (QIIME) software version 1.8.0^33^. The operational taxonomic units (OTUs) were picked based on sequence similarity within the reads. Taxonomy was assigned against the Greengenes database (version 13_8). The Uclust method with the Greengenes reference database (version 13.8) was applied for clustering the high-quality clean reads into OTUs with sequence similarity ≥ 97%^34^. The alpha and beta diversities metrics for each sample were calculated. To display the output data and graphically illustrate the differences between samples, a principal coordinate diagram (PCo) was created. The unweighted pair group method using UPGMA was used to establish the phylogenetic tree using MEGA-X, and the tree was visualized using ITOL v.5^35^. The microbial communities' predicated ecological and metabolic functions were mapped from taxonomy using Python's versatile script (collapse_table.py) using the FAPROTAX database^36^. All statistical studies and visualization were conducted by R version 3.60^37^.

### Statistical analysis of observed data

The statistical analyses of the data were performed by running GraphPad Prism version 5.0 (USA). The acquired values were expressed as the mean value ± standard deviation (STDEV). A two-way analysis of variance (*ANOVA*) was performed to define the significance between biofilm cell numbers and biofilm ages (days). Differences between values were assumed to be statistically significant at a p-value < 0.05. All experimental operations were performed in triplicate independently.

### Ethical approval

This article does not contain any studies with human participants or animals performed by any of the authors.

## Results

### Performance of MFC systems and electrogenic activity of biofilm

**Figure 2:**
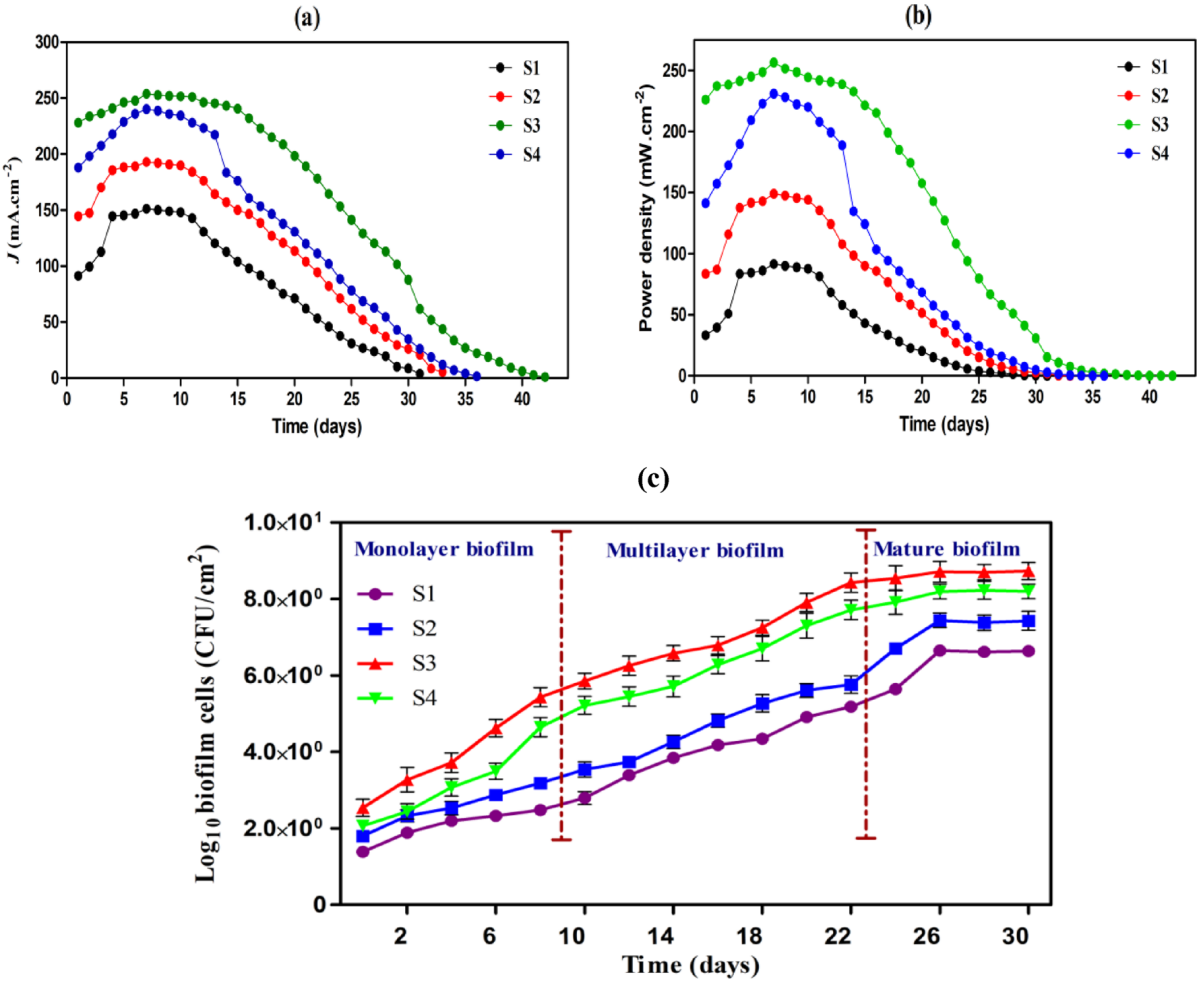
(**a**) Current density, (**b**) power density outputs as a function of time (days) with 1 K Ω as an external resistance in the MFCs constructed with different anodes (MFC-S1, MFC-S2, MFC-S3, and MFC-S4) using activated sludge as fuel, (**c**) Quantitative analysis of the growth rate of biofilm cells over different electrodes. Each data point is the mean of n = 3 at *P* value = 0.0001. The cell densities are calculated per electrode's unit surface area (cm^2^).

Some physicochemical characteristics of the activated sludge used as fuel and microbial sources for the present investigation were as follows: pH 7.12, COD 2015 mg O_2_ L^−1^, electric conductivity 4492 µS cm^−1^, total dissolved solids 2247 mg L^−1^, organic content 65%, and inorganic content 35%. To create the biofilm in a short time, two natural biomaterials, namely, potato powder and soybean powder, were explored, as these materials are known to enrich with high content of carbohydrates (15–20%) and protein (36–58%), respectively that are proper nutrient for rapid bacterial growth. The batch cycle operation at an external resistance of 1 K was employed to investigate electricity generation in the four MFC systems. After 42 days of microbial cultivation, the four MFCs established a repeatable cycle of current density and power density (Fig. 2a,b), showing the creation of biofilms and bacterial adherence had reached a steady state on the anode electrodes. The electrical performances of the MFCs with the biofilm-grown anodes (S1, S2, S3, and S4) in terms of current density (mA cm^−2^) and power density (mW cm^−2^) production in activated sludge water were examined. The maximum current output (mA cm^−2^) with the anodes S1, S2, S3, and S4 reached in seven days and were 151, 193, 324.2, and 248.75, respectively. The order of current production was MFC-S3 > MFC-S4 > MFC-S2 > MFC-S1. The highest current density of 324.25 mA cm^−2^ was gained in the MFC-S3, which was ~ twofold more elevated than the current (151.2 mA cm^−2^) generated in MFC-S1 (Fig. 2a). Furthermore, The results illustrated in Fig. 2b displayed that the maximum power density was produced by MFC-S3 system, with a value of 256.4 mW cm^−2^, followed by 230.8, 148, and 91.5 mW cm^−2^, respectively, for MFC-S4, MFC-S2, and MFC-S1 systems.

Results illustrated in Fig. 2c depicted the biofilm growth on four different anodes (S1-S4). As evident from the figure, the level of biofilm cells increased over time in all the electrodes, where the microbial density reached its maximum after 26 days of incubation. The biofilm accumulation was much more significant over S3, followed by S4, S2, and S1, where enormous numbers of biofilm cells were hooked over the modified anode compared with unmodified. The populations of biofilm cells discerned on the 26th day of incubation in S1, S2, S3, and S4 were 6.65 ± 0.24, 7.4 ± 0.31, 8.75 ± 0.18, and 8.19 ± 0.29 log CFU/cm^2^, respectively. We propose that the physicochemical property of PANI, as mentioned above, is a better parameter in the present case that surpasses the conductive properties of these graphene-based materials to induce higher electrogenic behaviors of the biofilm and linked current generation in the MFC. The preferential cultivation of electrogenic bacteria that can facilitate electrogenic activity and the transport of electrons to a concrete anode is achieved by anodic biofilm that has been habituated and is operated in closed-circuit environments^8^.

### Microscopic analysis of the electrogenic biofilm

**Figure 3:**
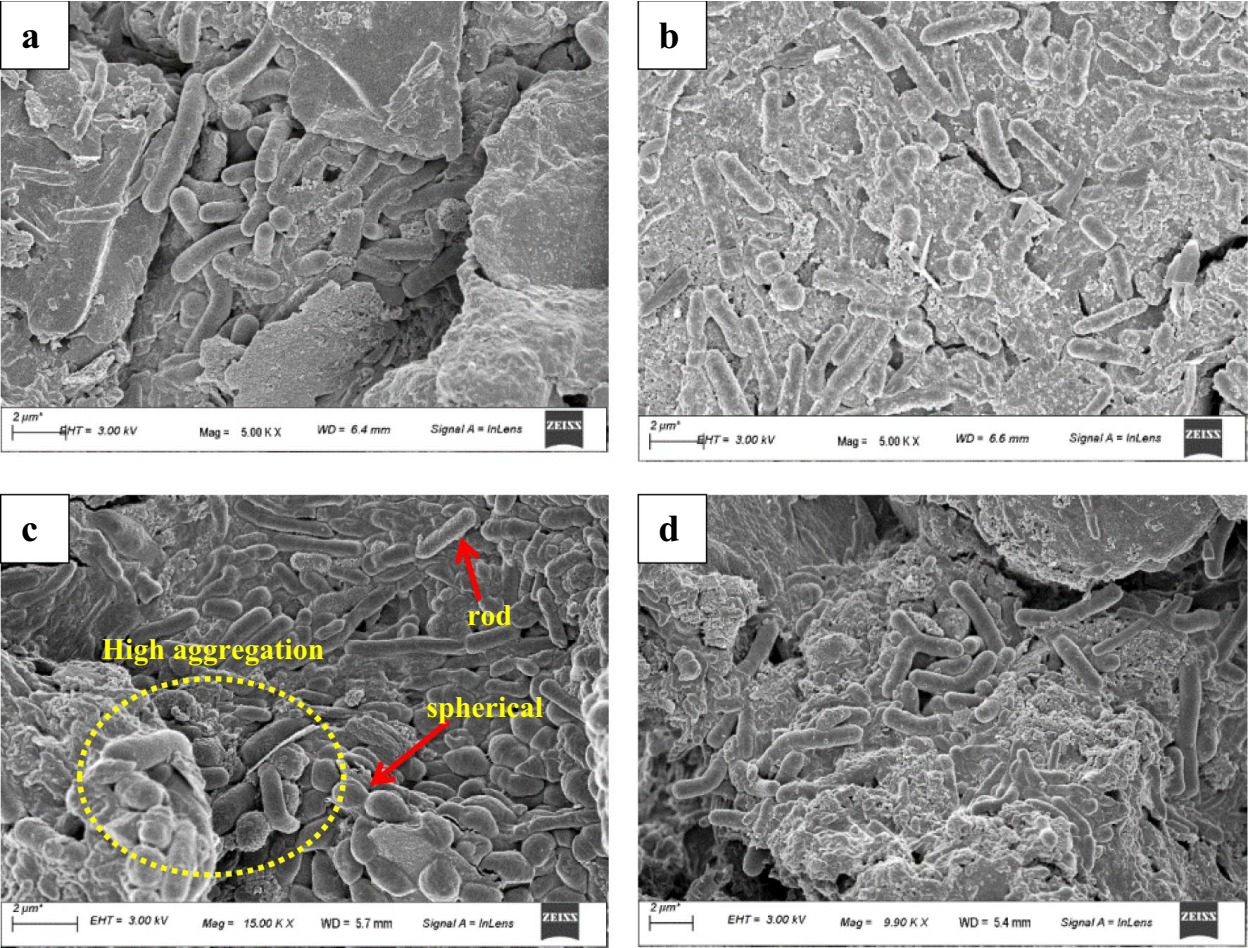
FESEM micrograph images of the morphology of anodic biofilm taken after 45 days of operation from (**a**) MFC-S1, (**b**) MFC-S2, (**c**) MFC-S3, and (**d**) MFC-S4.

FESEM microphotographs of the biofilm developed over the anode surfaces at the end of the operation period are presented in Fig. 3. Results pointed out that the biofilm formed on the electrodes was significantly wrapped in the coated matrices with different textures and cell population densities. At the MFC-S3 system, the whole anode surface, coated with PANI and plant powders, was covered with highly dense and thick biofilm. Moreover, the biofilm formed in the MFC-S3 system had a diverse morphological appearance as it contained different shapes of bacterial cells and included rod and round shapes (Fig. 3c). In the rest of the modified electrodes (S2 and S4), even though the bacterial cells of different shapes and sizes were visible, the overall cell density was significantly thinner than the S3 (Fig. 3a–d). Results reported the aggregation in MFC-S3 was more significant than the other coated and uncoated anodes. The biofilm formed on the uncoated graphite anode (S1) was much lesser than the other electrodes in terms of thickness and heterogeneity (Fig. 3a–d). Electroactive microorganisms occurring in the complex matrix of the electrode biofilm behave as a biological electrocatalyst in MFCs, generating current, as shown in Fig. 3. Such densely compacted biofilm cells can easily produce plenty of electrons by degrading the organic substances, resulting in a significant bioconversion efficiency of chemical energy stored in the anolyte solution. More substantially, the positive charge on the coated conductive materials encouraged electrocatalytic activities engagement with the negatively charged biofilm cells that support the speedy adherence and colonization of the electrogenic bacteria from the anolyte (wastewater) over the anode surface.

### Microbial community structure of the electrogenic biofilm

The microbial community analysis of electrogenic biofilm was performed by metagenomic analysis through High-throughput sequencing of the 16S rRNA gene to investigate the abundance and diversity of electrogenic bacterial biofilm. For the quantification of cells in the biofilm, the plat count method was considered^30^. The microbial community profile in the electroactive biofilm, formed at the end of MFCs operation on the fabricated anodes, was investigated as described in the following sections.

#### Microbial richness and diversity

**Figure 4:**
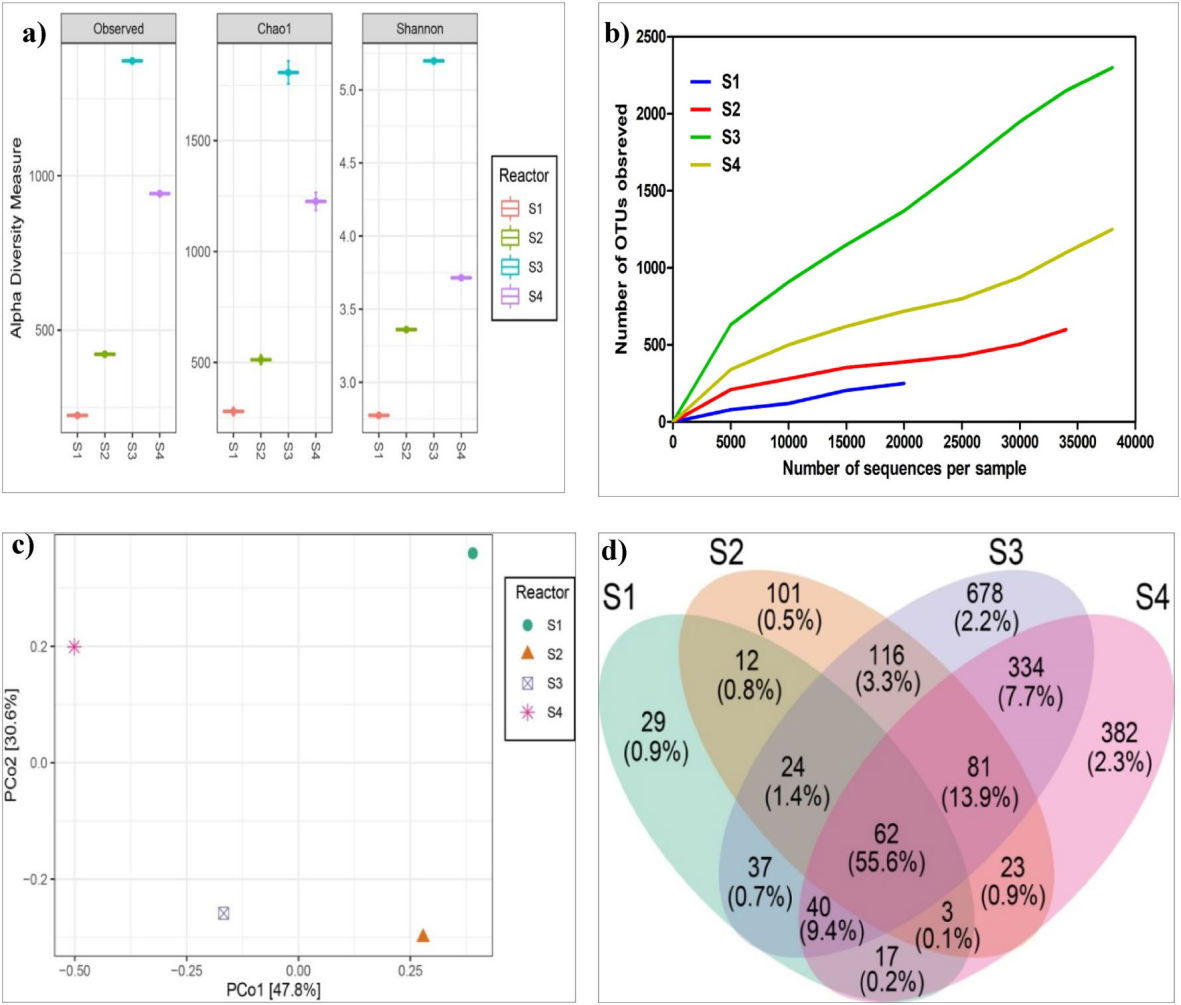
(**a**) Observed OTUs, Chao, and Shannon of anodic biofilm that developed over different anode surfaces. (**b**) Rarefaction curves of anodic biofilm formed over other fabricated anodes (S1–S4). (**c**) PCo plot shows the relationship among the biofilm communities. (**d**) Venn diagram of shared and uniquely identified the OTU of electroactive biofilm enriched with different energy substrates. The OTUs were defined at 0.03 distances.

From the sequence analysis of 16S rRNA genes, the reads generated from the S1 to S4 samples were recorded, as shown in Fig. 4a. The observed OTUs were identified at 97% nucleotide. The observed OTUs, Ace, and Chao1 estimators revealed that the anode biofilm of S1 reactors had the lowest while S3 reactors had the highest microbial abundance. The Simpson's diversity increased from 0.855 in the S1 reactor to 0.982 in the S3 reactor. The taxonomic abundance of OTUs levels in S1 vs S2 vs S3 vs S4 was 273, 572, 2085, and 1334. Similarly, Shannon's and Simpson's diversity indices were also the greatest for S3 biofilm. Generally, the microbial diversity indices were highest in the S3 biofilm compared to the other electrodes suggesting that the coating composite of plant mixture and PANI are pleasant to the formation of microbial community structure on the electrode. The alpha diversity indices indicated that the diversities of all the anodic communities are at different levels. The bacterial communities in S1 and S2 reactors were lower than those of the anodic biofilm of the S3 reactor.

The slopes of rarefaction curve analysis of the anodic biofilm samples revealed that the microbial community compositions in S3 reactors are vastly diverse from the S1 reactor, and the level of diversity increases in the order S3 > S4 > S2 > S1 (Fig. 4b). Although all the reactors were operated with the same inoculum of activated sludge, the microbial communities of the electrodes (S2-S4) were significantly distinct from those of the unmodified anode (S1), as revealed from the principal coordinates analysis (PCoA) at the genus level (Fig. 4c). The beta diversity analysis through the PCo plot, which provides the information on microbial community structure (the taxonomy of species) difference among the biofilm samples in habitats, indicates that the community structure of anodic biofilm in S3 was closest to S4. The scenario could be best represented through the Venn diagram, which was applied to compute the number of identical and unique OTUs in the four separate biofilm S1–S4, and illustrated the level of similarity and overlap in the OTU composition of the samples (Fig. 4d). It is evident from the data that S3, with 678 OTUs, had the most, followed by S4, with 382, S2, with 101, and S1, with 29 OTUs. It is worth noting that the PANI-modified anodes (S3) had a noticeable impact on bacterial community composition, expanding the number of OTUs in the communities and promoting unique OTUs.

### Microbial community distribution of biofilm at different taxonomic levels

**Figure 5:**
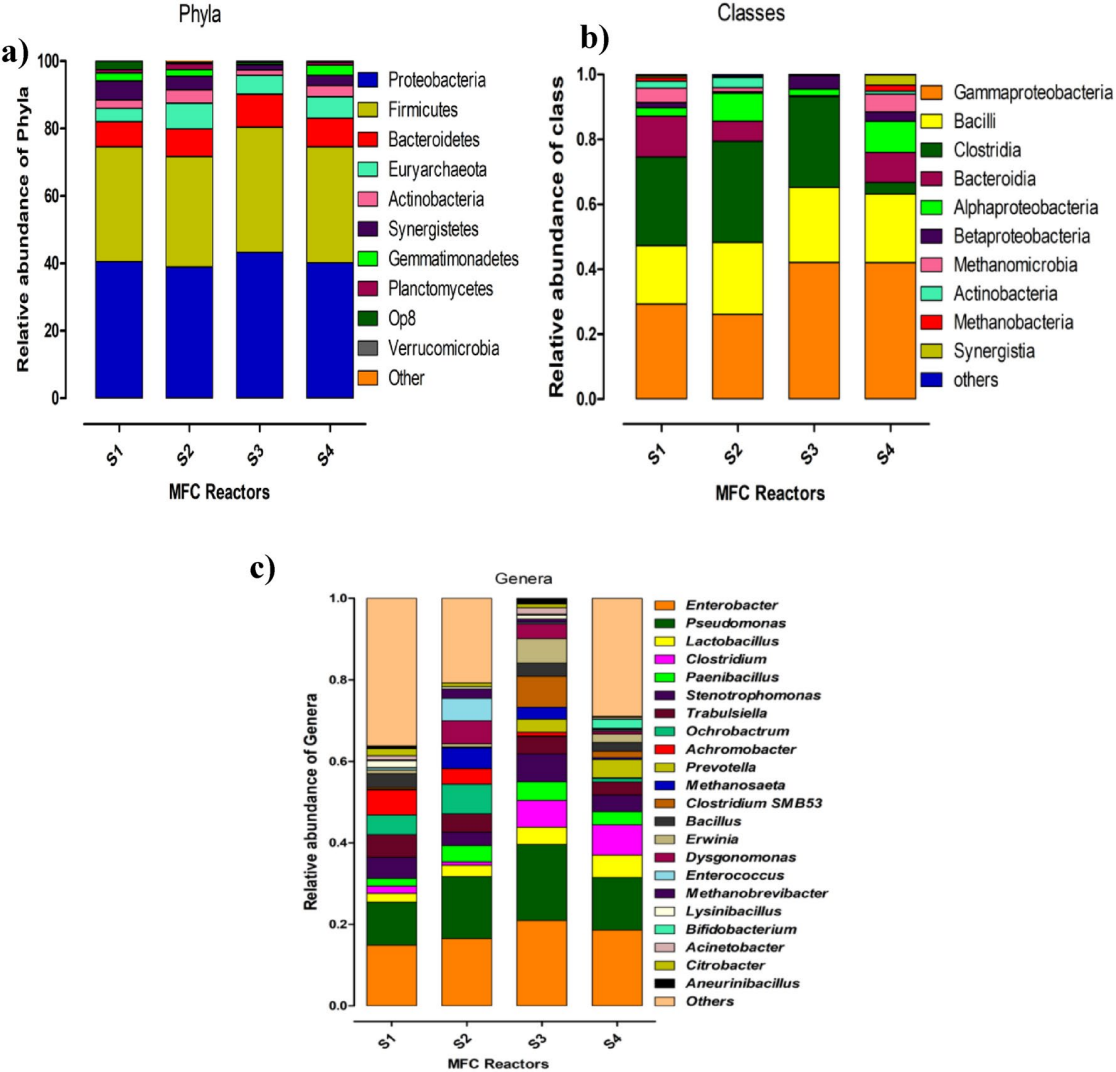
The relative abundance of the microbial community in the anodic biofilm developed over different anode electrodes (S1–S4) at phylum (**a**), class (**b**), and genus (**c**) levels.

The main phyla in four anodic biofilm samples were *Proteobacteria* (38.90%–43.21%), *Firmicutes* (32.76%–37.12%), *Bacteroidetes* (7.56%–9.82%), *Euryarchaeota* (3.85%–7.59%), and *Actinobacteria* (1.56%–3.99%) and the ranges of their relative abundance in the biofilm are displayed in the parentheses (Fig. 5a and Table 1).

**Table 1:** Relative abundance (%) of the most prevalent bacterial phyla in the anodic biofilm.

Phyla	MFC bioanodes^a^			
	S1	S2	S3	S4
*Proteobacteria*	40.5	38.9	42.21	40.18
*Firmicutes*	33.99	32.76	37.12	34.35
*Bacteroidetes*	7.56	8.22	9.82	8.57
*Euryarchaeota*	3.86	7.59	3.6	6.29
*Actinobacteria*	2.54	3.99	2.56	3.34
*Synergistetes*	5.6	4	2.55	3.04
*Gemmatimonadetes*	2.43	2.01	0	3.01

^a^Graphite-based anodes used were as follows. S1: control (no coating); S2: composite suspension of plant powder and rGO; S3: composite suspension of plant powder and PANI; S4: composite suspension of plant powder and CNTs.

At the class level, the significant classes in all biofilm samples were *Gammaproteobacteria* (26–42%), *Bacilli* (18–23), Clostridia (3–31.2%), *Bacteroides* (2–12.6), *Alphaproteobacteria* (2–9.6%), and *Betaproteobacteria* (0.03–4.1%). In the S3 biofilm, the relative abundance (%) of *Gammaproteobacteria*, *Bacilli*, *Clostridia*, and *Betaproteobacteria* were 42, 23, 28, and 4, respectively (Fig. 5b), which are more than the S1 biofilm. Conversely, the proportions of *Bacteroides* and *Alphaproteobacteria* were far less in the S3 biofilm. The relative proportions of the bacterial composition at the genus level are shown in Fig. 5c. The topmost ten prevalent genera identified were *Enterobacter, Pseudomonas, Lactobacillus, Clostridium, Paenibacillus, Stenotrophomonas, Trabulsiella, Ochrobactrum, Achromobacter*, and *Bacillus*. The percentage of the *Enterobacter* genus in the biofilm collected from the coated electrode with PANI (S3) increased to 20.92% from 14.86% in the uncoated electrode sample.

**Figure 6:**
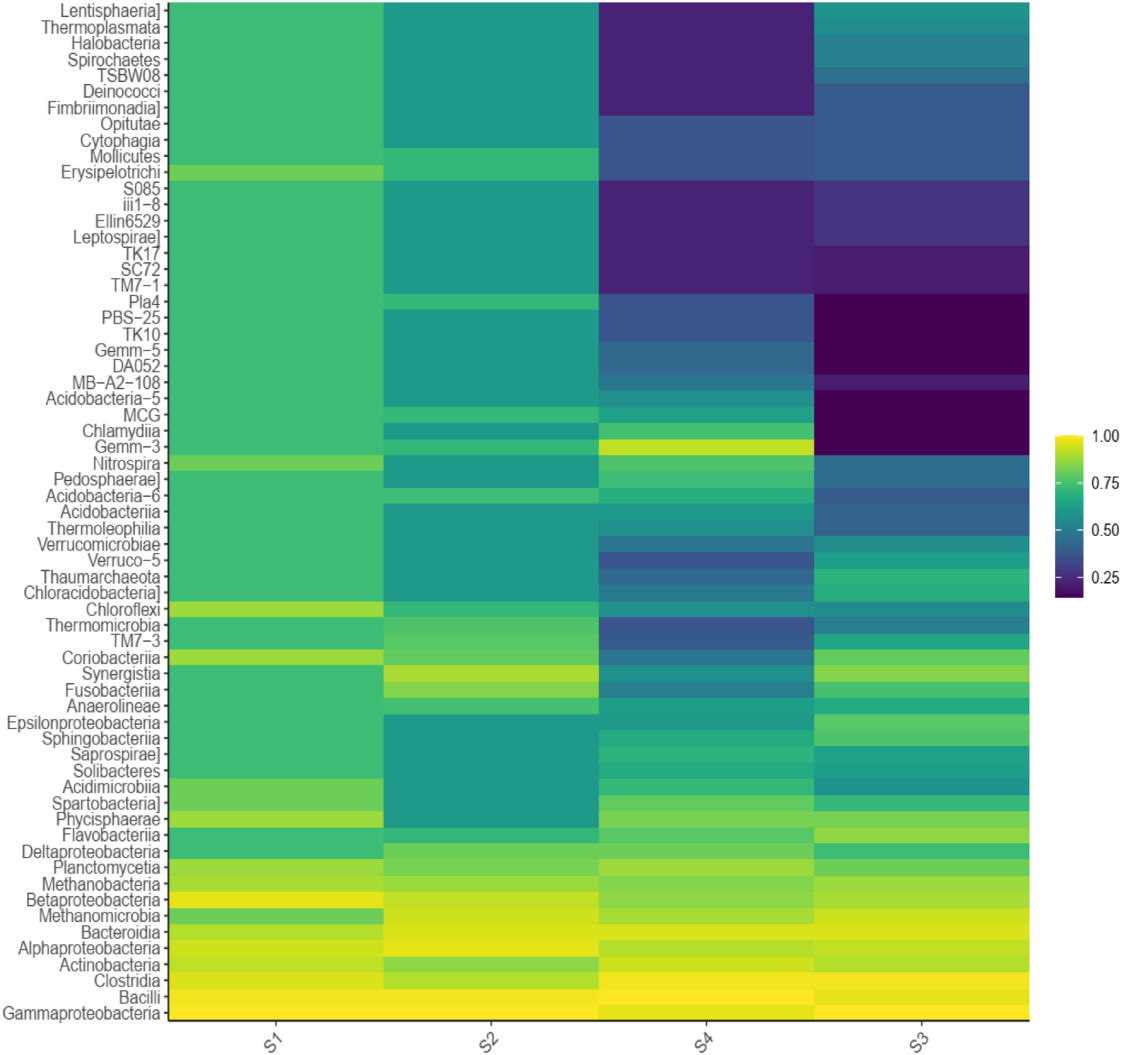
Phylogenetically clustered heat map of representative genera of microbial communities of anodic biofilm collected from S1–S4 biofilm. According to the color guide at the upper right, the intensity of color in each panel describes the relative abundance (%) of a genus in a sample.

The heat-map of with the cluster analysis was conducted to visualize the differences in the bacterial community structures at the genus level of the anodic biofilm samples. Data illustrated in Fig. 6 revealed the change of bacterial genera within the microbial assemblies of biofilm at the genus level. Following the operation of the MFC, the electroactive S3 biofilm was found to have more species than the other anodic biofilm. Results infer that a large number of electrogenic genera were observed in all the investigated anodic biofilm. In the S3 biofilm of, the highest relative abundances of *Enterobacter*, *Pseudomonas*, *Lactobacillus*, *Clostridium*, *Paenibacillus*, *Stenotrophomonas*, *Trabulsiella*, *Ochrobactrum*, *Achromobacter*, and *Bacillus*, were 20.92, 18.68, 4.21, 6.61, 4.55, 6.89, 4.19, 0.18, 0.94, and 3.21%, respectively*.* In contrast, their corresponding maximum relative abundances (%) in the S1 biofilm of S1were 14.86, 10.54, 2.19, 1.84, 1.80, 5.23, 5.59, 4.82, 6.16, and 3.36.

**Figure 7:**
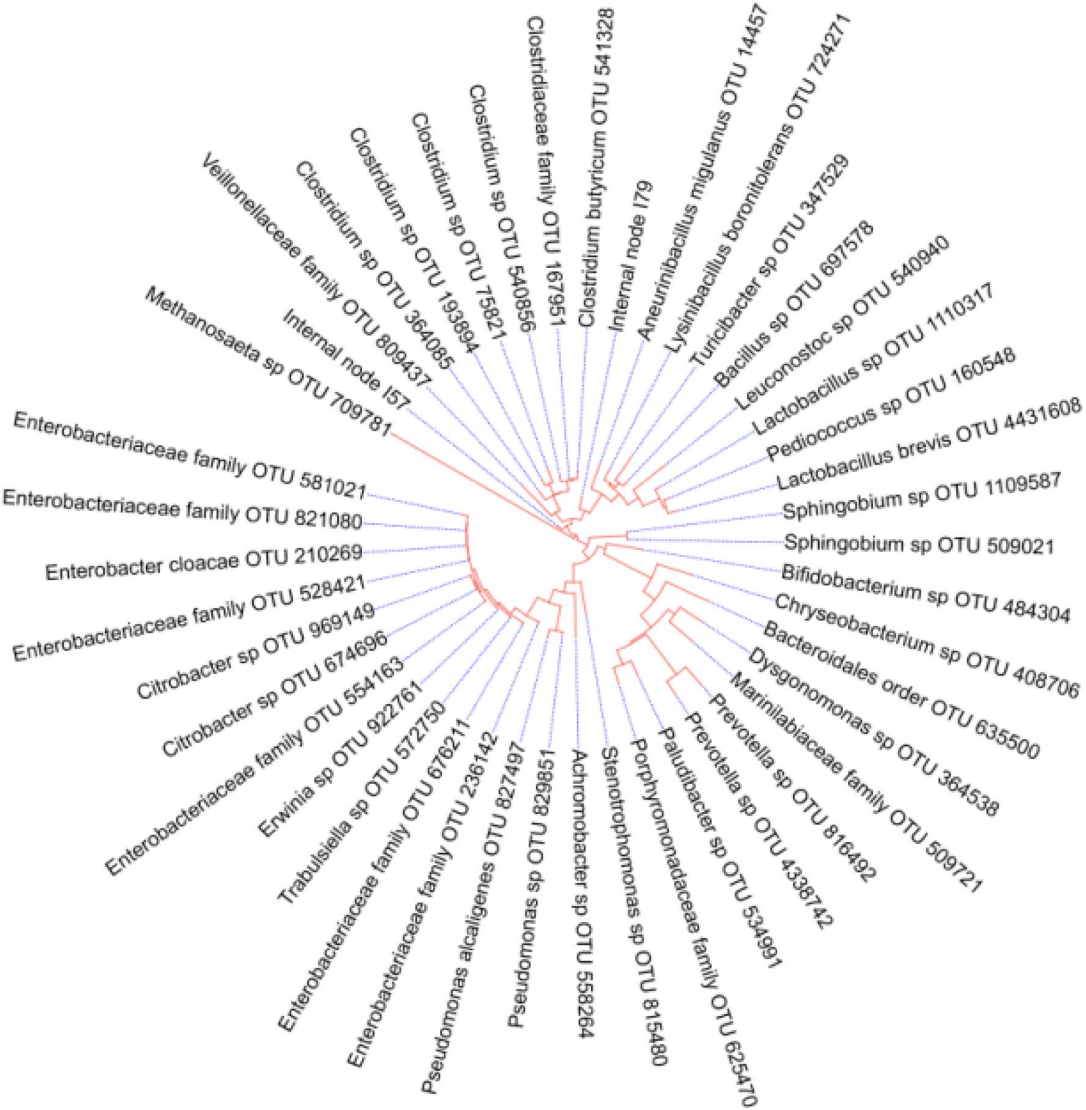
Phylogenetic analysis of distinctive electrogenic bacterial species used in air–cathode MFCs. A rooted phylogeniatc tree was consctructed using Mega-X software.

A phylogenetic assessment of the bacterial taxa in the anodic biofilm was performed following the 16S rRNA sequences analysis, as depicted in Fig. 7. It revealed the presence of many mesophilic bacterial species, the majority of which are known electrogens. As shown in the phylogenetic tree, it can be indicated that the anodic microbial biofilm revealed the potential association of some electrogenic bacteria, such as *Clostridium*, *Pseudomonas*, *Enterobacter*, and *Stenothermophilus*, in bioelectricity production.

### Functional profile in anodic biofilm

Based on the taxonomic information, the dynamics of the most abundant functional profiles for microbial communities of each anodic biofilm are illustrated in Table 2. In all the cases, the microbial functions related to carbon and nitrogen metabolism are visible with some variations in chemoheterotrophy, aerobic chemoheterotrophy, nitrification, nitrogen/nitrate/nitrite respiration, and nitrate/nitrate/nitrite denitrification. Some variations of sulfur metabolism and nitrate metabolism bacteria are also observed across all the biofilm. The presence of broader and more intense profiles in S3 compared to the biofilm of other anodes has been detected. Some of the major distinctions in the relative abundances of the functional groups in S3 from S1, S2, and S4 are indicated through the star symbol in Table 2. Among these distinctions, various types of methanogenesis functions, nitrate reduction, and sulfur respirations are prominent. The S3 biofilm thus preserved the critical functions of microbial populations, which are likely to support the degradation of organic waste in the activated sludge water under anoxic conditions. These functional profile variations and intensities within the anodes may be linked to their bioelectrochemical performance observed in the MFC.

**Table 2:** Functional profile among phylogenetic groups of microbial communities in anodic biofilm.

Functional groups	Relative abundant (%)			
	S1	S2	S3	S4
Methanotrophy	0	0.007	0.013	0.008
Acetoclastic methanogenesis	0.009	1.183	2.491	1.654
Methanogenesis using formate	0	0.025	0.347	0.012
Methanogenesis by CO_2_ reduction with H_2_	0.024	1.139	1.657	0.091
Hydrogenotrophic methanogenesis	0.024	1.141	1.507	0.095
Methanogenesis	0.034	3.324	5.996	0.452
Nitrification	0.004	0.014	0.063	0.035
Denitrification	0	0.139	0.184	0.113
Nitrate reduction	0.399	3.55	13.295	5.725
Sulfite respiration	0.006	0.012	0.068	0.031
Respiration of sulfur compounds	0.032	0.115	0.364	0.102
Reductive acetogenesis	0	0.0020	0.028	0.012
Aerobic ammonia oxidation	0	0.0211	0.0839	0.0621
Aerobic nitrite oxidation	0.048	0.021	0.132	0.497
Chemoheterotrophy	28.27	37.85	28.05	20.23

## Discussions

The superior electrical performance of the MFC with S3 anode has been attributed to the combined effect of PANI and biocompatible composite plant powder with the nutritive nature that induces massive growth of electrogenic biofilm on the graphite surface. Of note, the hostile nature of the bare graphite material hinders bacterial colonization over its surface^38,39^. Moreover, PANI in the composite is likely to help dock the bacterial species from the activated sludge water through electrostatic interaction due to the opposite charges of these interacting entities^40,41^. The results indicated that the anode (S3) coated with the plant powder containing the conductive polymer PANI contributed higher electrical current than the corresponding plant powder-coated rGO (conductivity of rGO: 3112 S cm^−1^) and CNTs (conductivity of CNTs: 106 to 107 S cm^−1^) to the MFC despite the extremely high conductivity of these last two types of nanomaterials as shown in the parentheses^14,42^.

The current production in the MFC gradually declined after reaching the maximum level following its operation for ~ 10 days. The main reason for such phenomena may be attributed to the gradual reduction of electrogenicity of the matured biofilm due to the continuing formation of the underneath death cell layer and nutrient limitations caused by the prolonged operation in batch mode^43^. We presume that most of these bacteria are endowed with the electrogenic property as the growth of these biofilm contributes electrical power to the constructed MFC. Notably, electroactive microorganisms (EAMs) are the kingpin of MFCs that function on the electrode surface to convert chemical to electrical energy through bioelectrocatalysis processes^44^. The preferential accumulation of electrogenic bacterial species, which could also regulate electrogenic behavior and the transmission of electrons to a rigid anode, are cultivated by anodic biofilm communities that have become habituated and are managed under closed-circuit settings^45^.

By using SEM visualization of biofilm, it can be indicated that the surface of the coated anode likewise appeared to be enveloped in a thick coating of extracellular polymeric substances discharged from bacterial activity. Rarely, such a polymeric covering layer may prohibit the biofilm's conductivity and restrict the transmission of electrons from microbes to the electrode surfaces. In contrast, the positive properties o this polymeric matrix is that it encourages the bacterial agglomeration and anode electrode biocompatibility. The polymeric covering subsance on the electrode surfaces may have assisted the observable microbial clustering (a concentrated bacterial community) there. The coated anode's excellent biofilm layer development contributes to the SMFC's low charge impedance^46^.

EAMs utilize organic compounds or carbon dioxide (in the case of autotrophic bacteria) and furnish electrons into the anode^47,48^. However, some non-electroactive microbes (non-EAMs) in the anodic biofilm may also contribute to improving the MFC performance by providing a suitable anaerobic environment and electron transfer mediators that redirect the electron path for the electrogenic microbes^6,49^. The electrical energy generated from the MFC is utilized for various purposes, such as wastewater treatment and biosensor applications^50,51^. The capability of EAMs to accumulate, acclimatize and propagate on the electrode surface influences the performance of the MFC-based electrochemical systems^52^.

This work revealed the microbial community pattern in the electrogenic biofilm developed over the modified graphite anode in an MFC setup operating with an activated sludge as the source of fuel and microorganisms. The electrogenic nature of the *Firmicutes* and *Proteobacteria* phyla, including *Alpha*, *Beta*, *Gamma*, and *Delta*^53–56^ and *Bacteroidetes* are known^57^. *Proteobacteria* are the most frequently reported phylum in anodic biofilm, followed by *Firmicutes* and *Bacteroidetes*^58,59^. However, the information on *Actinobacteria* and *Euryarchaeota* that are known to associate with the decomposing complex organics and alcohol^60,61^ and methanogenesis^62^, respectively, are not adequately understood. *Gammaproteobacteria* have been reported for their high electron transfer capability^63^, whereas, *Clostridia* are recognized for their potential to generate acetate pyruvate and hydrogen from carbohydrates^64^. Additionally, *Betaproteobacteria* and *Alphaproteobacteria* were abundantly found in MFC^65^. Further, *Enterobacter* (belonging to *Gammaproteobacteria*) is a common exoelectrogenic genus. It accounted for the highest proportion among all genera and thereby played a critical role in power generation^66^. The relative abundance of *Pseudomonas* in S3 biofilm was 18.675%. *Pseudomonas* belongs to *Gammaproteobacteria*. Gram-negative, facultative anaerobe produces electron transfer mediators, such as pyocyanin, Phenazines, and related substances that contribute to the MFC power^67–69^. The relative abundance of *Clostridium* decreased from 7.45% in the anodic biofilm (S4) sample to 0.82% in the uncoated anode (S1) samples. Definitely, *Clostridium* sp. has been progressively recognized as a dominating species in MFCs^11,70,71^. The relative abundance of *Lactobacillus* decreased from 5.47% in the coated anode (S4) sample to 2.185% in the uncoated anode (S1) sample. The role of *Lactobacillus* sp. on lactose fermentation, producing the electroactive biofilm, and generating bioelectricity from waste dairy water without a mediator are known^72^.

All the biofilm had a significant population of *Bacteroides* and *Dysgonomonas*. The relative abundance of *Dysgonomonas* increased to 5.61% in the S2 from 0.01% in the S1. The abundance of *Dysgonomonas* (belonging to *Bacteroidia*) was found to be associated with the increase in power output of an MFC^73^. The relative abundance of *Acinetobacter* increased to 1.56% in the S3 from 0.03% in the S1; they are described as fermentative bacteria for carbohydrate metabolism^74^. Moreover, *Acinetobacter* has been proposed as a significant component of the electroactive biofilm population^75^. Some previous investigations revealed that *Acinetobacter* spp. that utilizes H_2_ was predominant in MFCs^76^. The results endorse the existing hypothesis that a pluralistic microbial community as the anodic biocatalyst in MFCs contributes to improving electrical power from complex fuel sources^11^. The studies on the correlation between functional profiles and electrogenic behaviors of bacteria are limited. The exoelectrogenic behaviors of nitrate-reducing bacteria, such as *Pseudomonas,* and electrical current production may be linked to the anaerobic respiration pathways of fermentative bacteria, such as *Clostridium* and *E.coli,* have been documented^13,77^.

The difference among the fabricated anodes enacted by introducing different conductive materials did not drastically change the prominent profile of bacteria, as evident from the dominance of the phyla, *Proteobacteria, Firmicutes*, and *Bacteroidetes* in the biofilm across all these anodes. However, in the class and species levels, the variations were distinct and were highest in the later case. For instance, PANI modified anode exhibited the highest relative abundance of *Gammaproteobacteria*, *Clostridia*, and *Bacilli* at the class level and *Pseudomonas*, *Clostridium*, *Enterococcus*, and *Bifidobacterium*, at the species level. Likewise, the microbial taxonomic classes, which are significantly dominant, were *Gammaproteobacteria* and *bacilli*. Interestingly, as discussed above, the MFC produced a higher electrical current with PANI than rGO and CNTs as doping material to the composite powder on the anode, even though the latter two graphene-based materials possess better conductivity and are conventionally used to harvest electrical power in MFC^78^.

## Conclusions

Coating anode electrodes with conductive composites are well-practicing to promote biofilm formation and electrocatalytic reaction. This study revealed the microbial community pattern in the electrogenic biofilm developed over a modified graphite anode in an MFC setup operating with activated sludge. The difference among the fabricated anodes enacted by introducing different conductive materials drastically changed the prominent structural profile of the bacteria in the class and species levels, and the variations were higher in the latter case. The selection of coating material had a considerable impact on microbial diversity, whereas the biofilm collected from MFC-S3 had substantially greater observed OTUs levels than other materials. For instance, PANI modified anode exhibited the highest relative abundance of *Gammaproteobacteria*, *Clostridia*, and *Bacilli* at the class level and *Pseudomonas*, *Clostridium*, *Enterococcus*, and *Bifidobacterium*, at the species level. These results displayed that the anodic biofilm's bacterial community structure could be significantly altered by changing the conductive materials on the graphite electrodes. Interestingly, the MFC produced a higher electrical current with PANI than rGO and CNTs as doping material to the composite powder on the anode. This study provides insight into the interaction between cells and the electrode and the growth dynamics of biofilm on the anode and provides a significant stimulus for developing MFCs technology for practical applications.

## Data Availability

The raw data used in this study were uploaded to the NCBI Sequence Read Archive (SRA) under Bioproject Accession Number PRJNA801836.
